# Oral cancer screening knowledge and practices among dental professionals at the University of Toronto

**DOI:** 10.1186/s12903-023-03062-3

**Published:** 2023-05-31

**Authors:** Dorsa Mavedatnia, Karl Cuddy, Hagen Klieb, Nick Blanas, Jade Goodman, Melanie Gilbert, Antoine Eskander

**Affiliations:** 1 Faculty of Medicine, University of Ottawa, Ottawa, ON Canada; 2 Division of Oral and Maxillofacial Surgery, University of Toronto, Mount Sinai, Princess Margaret and Humber River Hospitals, Toronto, ON Canada; 3 Department of Dental and Maxillofacial Sciences, Sunnybrook Health Sciences Center, University of Toronto, Toronto, ON Canada; 4 Faculty of Dentistry, University of Toronto, Toronto, ON Canada; 5 Department of Otolaryngology - Head & Neck Surgery, Surgical Oncology, University of Toronto, Sunnybrook Health Sciences Centre and Michael Garron Hospital, Toronto, ON Canada; 6 Institute of Health Policy, Management, and Evaluation, University of Toronto, Toronto, ON Canada

**Keywords:** Oral cavity cancer, Screening, Dental professionals, Dentist, Oral cancer, Knowledge

## Abstract

**Introduction:**

Opportunistic oral cancer screening during visits to the dentist is a non-invasive and accessible option for detection of pre-malignant lesions and early-stage malignancies. The objective of this study was to investigate the knowledge, practices, and attitudes towards oral cancer screening among dentists.

**Methods:**

A 42-item survey was sent to 650 dental professionals affiliated with the University of Toronto. Data regarding training/practice characteristics, knowledge of oral cavity cancer, current screening practices, attitudes towards screening, and remuneration were collected.

**Results:**

Ninety-one dentists responded. Most obtained their dental degree from Canada (71.4%) and were practicing in large urban centers (87.9%). Most dentists correctly identified the oral tongue (87.8%) and floor of mouth (80%) as the two of most common sites of oral cavity cancer but only 56% correctly identified the most common presentation. 91% performed intra/extra oral examinations at every patient visit. Only 9.9% of dentists discussed the risk factors of oral cancer and 33% were not familiar with resources for smoking cessation and alcohol abuse. International medical graduates were more likely to discuss risk factor management than Canadian medical graduates (*p* < 0.01). Over 80% of dentists referred to a specialist when a suspected lesion was found. The greatest barrier for oral cancer screening was lack of time. Almost all dentists (98.8%) reported that their screening practices do not differ depending on the patient’s insurance status and 63.8% reported compensation would not influence their decision to perform oral examinations.

**Conclusion:**

Most dentists have a good knowledge of the presentation and risk factors associated with oral cavity cancer. Most dentists perform screening with every patient, with no influence from compensation and insurance status. Dentists are therefore an excellent first contact for oral cavity cancer screening for the general public and for high-risk populations.

**Supplementary Information:**

The online version contains supplementary material available at 10.1186/s12903-023-03062-3.

## Introduction

Oral cavity and oropharyngeal cancer have the sixth highest incidence rate of all cancers globally [1]. Oral cancer has a ~ 50% mortality rate and accounted for 177,000 global deaths in 2020 [2, 3]. In Ontario, despite decreases in oral cavity cancer incidence and increases in 5-year survival rates, oral cavity cancer still has a mortality rate of 2.9 deaths per 100,000 individuals based on 2020 data [4, 5].

Early diagnosis of oral cancer is associated with improved overall survival; however, most cases present with advanced disease [6]. Oral cancer is detected at early stages in only 30% of cases, and survival rates can improve by 50% when it is detected at localized stages [7]. Early diagnosis is not only the most effective way to improve quality of life and decrease morbidity and mortality, but it can also reduce therapy-related limitations in speaking, eating, and swallowing for patients [8, 9].

Visual examination of the oral cavity for screening of cancer allows for early detection of malignant and pre-malignant lesions that is time-efficient, painless, and non-invasive [10, 11]. Dentists are increasingly involved in the detection of oral cancer and precursor lesions [10]. During routine interactions with their patients, dentists have an opportunity to intervene and perform opportunistic oral cancer screenings [11–14]. Literature has suggested that dental based providers detect oral squamous cell carcinoma at earlier stages than physician colleagues [15]. Oral cancer screening leading to early diagnosis and improved mortality, a gap in knowledge and practice has been demonstrated amongst dentists, suggesting underutilisation of comprehensive oral cancer screening in practice [16–22].

There is limited data assessing the knowledge, attitudes, and practices of dentists regarding oral cancer screening. Previous studies conducted in Ontario have examined the role of oral and maxillofacial surgeons in the management of oral cavity cancer, the involvement of dentists in the diagnosis of oral cavity cancer, the role of dental hygienists, and barriers faced by dentists in screening [10, 23–25]. The objective of this study was to assess (1) the knowledge, barriers, and attitudes towards screening of oral cancer among dentists, (2) current practices of oral cancer screening and remuneration patterns, and (3) to compare screening practices among Canadian medical graduates (CMGs) and international medical graduates (IMGs).

## Methods

This study was a prospective cross-sectional survey distributed to community dentists and dental hygienists affiliated with the University of Toronto, Department of Dentistry. This study received ethics approval (REB#5135) from Sunnybrook Health Sciences Center at the University of Toronto. The Checklist for Reporting Results of Internet E-Surveys (CHERRIES) criteria was followed in reporting the survey study findings [26].

### Survey design and distribution

The survey consisted of 42 Likert-type and close-ended questions in 4 sections: demographics and career characteristics (5 items), knowledge of evidence around screening (3 items), current practices (17 items), attitudes towards screening (10 items), and remuneration (7 items) (Appendix I). It was developed using previously published studies which assessed the attitudes and practices of dentist with regards to oral cancer as reference [27–31]. Modifications to the questions were made to suit the sampled population.

Demographics encompassed gender, years in practice, specialty, and location of training, which was classified as CMGs and IMGs. The knowledge section assessed awareness of risk factors and common presentations, the current practices section assessed the frequency and comprehensiveness of oral examinations, motivators for screening, follow-up procedures, and management of suspicious lesions, and the attitudes section assessed barriers to screening, confidence, and desire for further training. The remuneration section characterized variations in billing and reimbursement.

The content and face validity of the questions was confirmed by two senior authors (AE, KC). Prior to distribution, the survey was tested among a convenient sample of 5 dentists to ensure ease of completion, clarity of interpretation, face validity, technical functionality, and time required for completion.

The closed survey was distributed by the study investigators through the Department of Dentistry between January to March 2022 via e-mail; a total of 3 e-mails were sent approximately 1 month apart. Participants were provided with 1 month to complete the survey after the last e-mail was sent. SurveyMonkey, an online platform, was utilized to capture responses. There was no tracking method for unique participants. Participants were provided with the length of the survey, the time required to complete the survey, where data would be stored, the investigators involved in the study, and the study’s goals and purposes. Informed consent was obtained through participation in the survey and participants were informed that participation is voluntary and of their responsibilities. The data was anonymous, no identifiable information was collected, and no incentive was provided for completing the survey. There were 6 pages: demographics (5 questions), knowledge of risk factors (3 questions), current practices (17 questions), attitudes (10 questions), and reimbursement (7 questions). The survey did not include a completeness check and participants had the ability to review and change their answers.

Convenience sampling was utilized. The mailing list included all dentists and dental hygienists affiliated with the University of Toronto, Department of Dentistry through teaching or education, including community affiliated members. Recruitment included 100 dental professionals with academic affiliations and 550 instructors. Recruitment of CMGs and IMGs was not done separately and data was collected in the survey to encompass location of training.

### Statistical analysis

All questionnaires were analyzed. Descriptive statistics including mean, median, standard deviation and interquartile range were calculated where appropriate. A chi square test was performed to assess differences in practices between international medical graduates (IMG) and Canadian medical graduates (CMG). Statistical significance was defined as a two sided *p*-value < 0.05.

## Results

### Demographics

The survey was sent to 650 dental professionals associated with the University of Toronto, with a response rate of 14% (*n* = 91). Three of the respondents were dental hygienists and 91 were dentists. Due to heterogeneity and small sample size, the dental hygienists were excluded from the analysis. Most of the participants were male (*n* = 56, 61.5%) and obtained their dental degree from Canada (*n* = 65, 71.4%) (Table 1). Majority of dentists were practicing in large urban centers (*n* = 80, 87.9%) and had > 20 years of experience (*n* = 47, 51.6%) (Table 1).

**Table 1:** Respondent demographics

	**Responses (*n* = 91) n (%)**
**Female**	35 (38.5%)
**Male**	56 (61.5%)
**Number of years in practice**	
< 5	12 (13.2%)
6–10	15 (16.5%)
11–20	17 (18.7%)
> 20	47 (51.6%)
**First dental degree acquired in Canada**	65 (71.4%)
**First dental degree acquired in other country**	26 (29.6%)
**Setting of practice**	
Small population centers (1,000–29,999)	5 (5.5%)
Medium population centers (30,000–99,999)	6 (6.6%)
Large urban centers (100,000 +)	80 (87.9%)

### Knowledge of risk factors

Most dentists correctly identified the oral tongue (*n* = 79, 87.8%) and the floor of mouth (*n* = 72, 80%) as the two of most common sites of oral cavity cancer. Smoking, betel nut, and alcohol were correctly identified as the most common risk factors for oral cavity cancer by 98.9% (*n* = 89), 75.6% (*n* = 67), 65.6% (*n* = 59) of dentists, respectively. The most common presentation of oral cancer (a non-healing ulcer) was correctly identified by 55.6% (*n* = 50) of dentists.

### Current practices

**Fig. 1:**
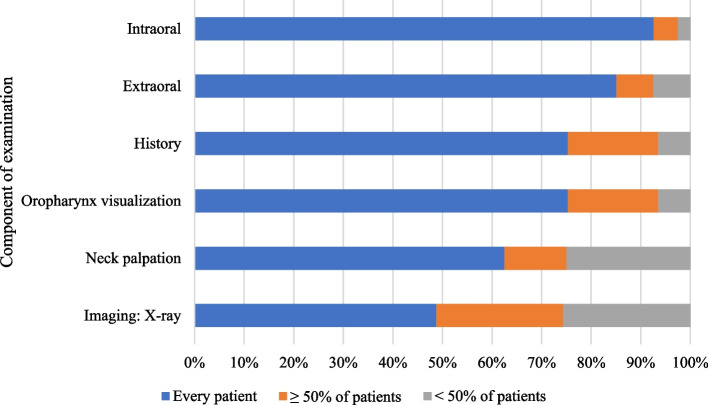
Percentage of dentists who perform each component of the oral cancer screening examination. Note: intraoral examination was listed as referring to: components of the oral cavity. Oropharynx was listed as referring to: tonsils and soft palate

Majority of dentists (*n* = 70, 87.5%) believe that oral cancer screening through visual examination is an effective method for its early detection. In dental offices, 49.4% (*n* = 40) reported that only dentists are responsible for oral cancer screening, whereas 50.6% (*n* = 41) reported that both dentists and dental hygienist perform oral cancer screening. Intra/extra oral examinations were most frequently performed in every patient in new patient appointments (*n* = 74, 91.4%), followed by asymptomatic patients (*n* = 61, 75.3%), and follow-up patients (*n* = 49, 60.4%) (Table 2). The most frequently reported component performed for oral cancer screening was the intra oral exam (*n* = 75, 92.6%) (Fig. 1). Only 9.9% (*n* = 8) of dentists discussed the risk factors of oral cancer with every patient and 12.4% (*n* = 10) counselled on risk factor management (Table 2).

**Table 2:** Oral cancer screening practices at patient appointments

	**Responses (*n* = 81)—n (%)**		
	Every patient n (%)	≥ 50% of patients n (%)	< 50% of patients n (%)
**Oral examinations (intra/extra oral)**			
New patient	74 (91.4%)	4 (4.9%)	3 (3.7%)
Follow up patients	49 (60.4%)	16 (19.8%)	16 (19.8%)
Asymptomatic patients	61 (75.3%)	11 (13.6%)	9 (11.1%)
**Discussion of risk factors**^**a**^	8 (9.9%)	28 (34.5%)	45 (55.6%)
**Counselling of risk factors management**	10 (12.4%)	30 (37.0%)	41 (50.6%)

^a^Discussion refers to: screening for risk factors during history taking

### Dentists – no oral cancer screening

In dentists who did not perform oral cancer screenings for all patients (*n* = 49), 61.2% (*n* = 30) reported that it was not necessary for all patients, 36.7% (*n* = 18) reported it was due to time constraints, 4.1% (*n* = 2) reported they were not trained enough, and 2.0% (*n* = 1) stated it was not effective.

**Fig. 2:**
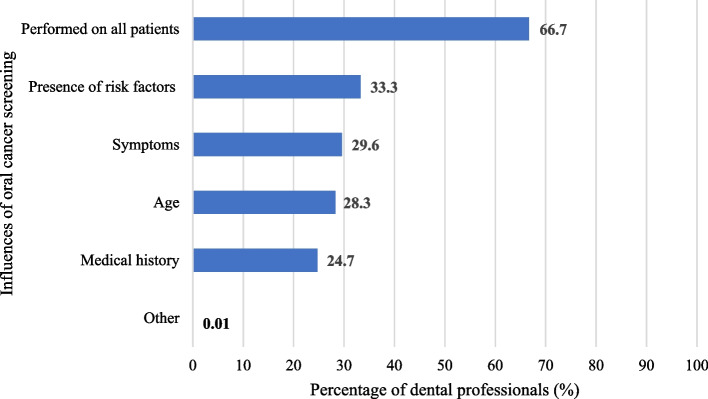
Motivators of oral cancer screening

The greatest motivator for oral cancer screening amongst dentists who do not perform screening on all patients (*n* = 27) was the presence of patient risk factors (*n* = 27, 100%), followed by patient symptoms (*n* = 24, 88.9%), patient age (*n* = 23, 85.2%), and medical history (*n* = 20, 74.1%) (Fig. 2).

**Fig. 3:**
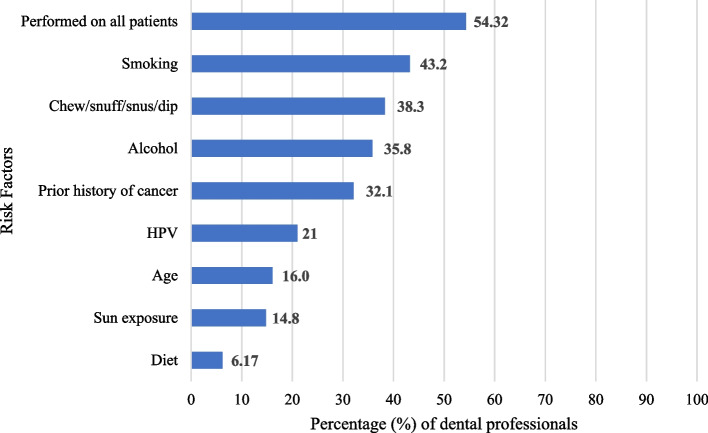
Risk factors that prompt oral cancer screening and examination

In dental professionals (*n* = 37) that do not perform oral cancer screening for all patients, the risk factors that prompted oral cancer screening were smoking (*n* = 35, 94.6%), chew/snuff/dip (*n* = 31, 83.8%), and alcohol (*n* = 29, 78.4%) (Fig. 3).

### Oral cancer screening – IMG vs CMG

There were 22 IMGs and 55 CMGs who responded to the question regarding frequency of screening. IMGs were not (20/22; 91%) more likely to screen for oral cancer at every visit compared to CMGs (54/55; 98%, *p*-value = 0.13). However, IMGs were more likely to discuss risk factors (43%) at every visit compared to CMGs (5%; *p*-value < 0.01).

### Management

Most dentists (*n* = 59, 72.8%) spent 1–5 min performing intra/extra oral examinations; 9.9% (*n* = 8) spent < 1 min and 17.3% (*n* = 14) spent > 5 min. When asked regarding the management of suspected lesions, 81.50% (*n* = 66) directly referred to a specialist, 55.6% (*n* = 45) utilized photo documentation of the lesion, 49.4% (*n* = 40) biopsied the lesion, 38.3% (*n* = 31) scheduled a follow up appointment, 25.9% (*n* = 21) utilized imaging modalities (x-ray), 22.2% (*n* = 18) asked the patient to self-monitor, and 3.7% (*n* = 3) instructed the patient to contact a physician on their own. While 32.1% (*n* = 26) of dentists were not familiar with resources for smoking cessation and alcohol abuse programs to provide to patients, 42% (*n* = 34) were interested in receiving greater training on tobacco and alcohol cessation programs.

### Attitudes and self-perceived knowledge

Almost all dentists (*n* = 69, 86.3%) believed that they received sufficient training to detect lesions suspicious of oral cancer, while 13.7% (*n* = 11) believed that they did not. Dentists expressed they were confident that their current knowledge of oral cancer is up to date (*n* = 38, 47.5%), in detection of oral cancer (*n* = 40, 50%), and their ability to perform a comprehensive oral examination (*n* = 32, 40%) (Table 3). Concerning how dentists would rate their knowledge on oral cancer detection and prevention, 72.5% (*n* = 57) rated their knowledge as good, 25% (*n* = 20) rated it as “satisfactory”, and 2.5% (*n* = 2) rated it as “poor”.

**Table 3:** Confidence of dentists towards components of oral cancer

	**Responses (*n* = 80) n (%)**			
n (%)	**Very confident**	**Confident**	**Satisfactory**	**Not confident**
Ability to perform comprehensive oral examination	42 (52.5%)	32 (40%)	5 (6.2%)	1 (1.3%)
Detect oral cancer	18 (22.5%)	40 (50%)	20 (25%)	2 (2.5%)
Knowledge on oral cancer	18 (22.5%)	38 (47.5%)	19 (23.8%)	5 (6.2%)

### Barriers to screening and education

**Fig. 4:**
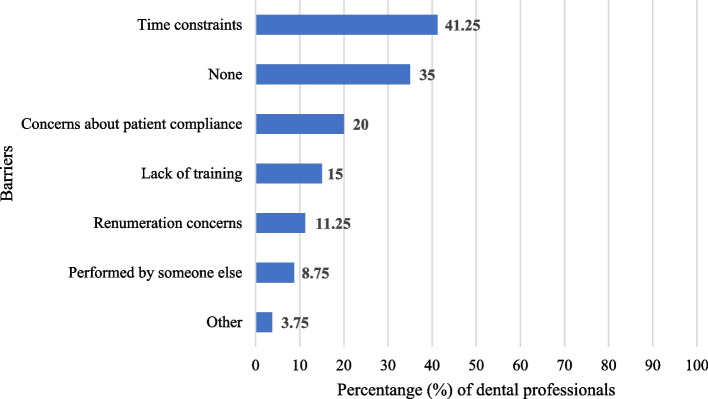
Reported barriers to oral cancer screening

While the greatest barrier to intra/extra oral examinations that dentists reported was the lack of time (*n* = 33, 41.3%), 35% (*n* = 28) of dentists reported no barriers as they performed screenings for every patient (Fig. 4). Half of dentists (*n* = 40) attended a continuing education course on the screening and management of oral cancer within the last 2–5 years; 21.2% (*n* = 17) attended < 12 months ago, 20% (*n* = 16) attended 5 + years ago, and 8.8% (*n* = 7) never attended a course. Most dentists (*n* = 62, 77.5%) had an interest in attending continuing education courses on the detection and counselling of oral cancer.

### Renumeration

Most dentists (*n* = 66, 82.5%) reported that they cannot bill insurance companies or the patient for oral cancer screening and 65% (*n* = 52) reported that billing for a routine examination at an initial appointment includes the oral cancer screening exam. Almost all dentists (*n* = 79, 98.8%) reported that their screening practices do not differ depending on the patient’s insurance status and 63.8% (*n* = 51) reported compensation would not influence their decision to perform oral examinations as they screen every patient.

Most dentists (*n* = 48, 60%) are unsure if they can bill for smoking and alcohol cessation. One fifth (*n* = 16) of dentists stated that they can bill for smoking/alcohol counselling, while one fifth (*n* = 16) stated that they cannot. One third (*n* = 24) of dentists reported they would not counsel patients on risk factor management more often if they were paid as they screen every patient.

## Discussion

This is the first study to assess oral cancer screening barriers and remuneration attitudes amongst dentists in Ontario. In this survey of 91 dentists from the greater Toronto area and affiliated with the University of Toronto, knowledge of the presentation and risk factors associated with oral cavity cancer was good. Most dentists believe that oral cavity cancer screening is effective and are performing it in their offices, independent from insurance policy or compensation as this activity is largely non-remunerated. The greatest reported barrier to screening is a lack of time. Over 75% of dentists were interested in attending a continuing education course on detection and counselling of oral cancer. Our work should therefore inform future continuing education events among dental professionals in Canada.

Oral cavity cancers present late, with significant impacts on morbidity and quality of life [32]. Opportunistic oral cancer screening during visits to the dentist or dental hygienist is a feasible, non-invasive, and accessible option for detection of pre-malignant lesions and early stage malignancies [11, 13, 14]. Visual screening for oral cancer not only improves detection, but can also reduce mortality in high-risk individuals [33, 34]. Oral cancer screening is also cost-effective, particularly in high-risk populations, and dental providers can detect oral squamous cell carcinoma at earlier stages than physician colleagues [15, 35, 36]. In a previous study assessing the opinions and attitudes of dental professionals in the United Kingdom on oral cancer screening, almost all participants strongly believed that visual screening is effective for the early detection of cancer and that a national-based screening program would be effective to improve morbidity and mortality of oral cancer [37]. Similar results were found in other studies. Dentists employed in the public system in Brazil recognized the importance of preventative measures for oral cancer and over 95% of respondents performed full mouth examinations for screening of oral cavity cancer [38]. As it relates to screening, dentists are typically the first health care professionals to examine the oral cavity, which provides an optimal opportunity to screen for oral cancer [39, 40]. Oral cancer screening by dentists appears to be time effective as majority in this study reported spending between 1–5 min on oral exams. Most dentists were found to refer to specialist or perform a biopsy when a suspicious lesion was detected. This is not unreasonable as biopsies are often not performed by general dentists but by oral and maxillofacial surgeons, oral pathologists, or oral medicine specialists and often reviewed by oral pathologists. While many general dental offices do not accept governmental insurance plans, such as Ontario Disability Support Program, specialists work without directly billing the patient. Financial barriers likely play a small role in specialist referrals, however, regional barriers may exist as fewer dental specialists exist in remote and rural areas compared to urban regions. Over 90% of dentists in this study were performing oral cavity cancer screenings in new patients, compared to a previous studies conducted in 2019 and 2017 reporting 43.6% of dentists in Japan and 51% in Australia [17, 18]. From the patient’s perspective, oral cancer screening is welcomed and well-perceived [41]. While they are generally unaware of oral cancer screening being performed, they are happy to take part in the screening processes and would like to be informed regarding the signs of oral cancer [41]. Patients would also like help from their dentist to reduce their risk of developing oral cancer [41].

The greatest risk factors for oral cavity cancer are tobacco and alcohol use, both of which are modifiable. This suggests that counselling may play an important role in improving outcomes in this population. In this cohort, only 12% of dentists were providing risk factor counselling at patient appointments. Alcohol and smoking were found to be the greatest risk factors prompting dentists to perform screening. Also, IMGs were found to be more likely to discuss risk factors with their patients. There is a high prevalence of oral cavity cancer in Asian countries and particularly in Southeast Asia, which could explain why IMG-trained dentists are more likely discuss risk factors given higher use of these substances internationally [42, 43]. Interestingly, patients from Southeast Asia, are considered a high risk population even when they are second generation immigrants and therefore may warrant additional screening even in the North American context [44].

Most dentists in this survey were interested in attending a continuing education course for oral cavity cancer screening. Similarly, a cross-sectional survey of dentists in Yemen found that majority believed they need greater training in oral cancer screening, suggesting that continuing educational programs for the early prevention and detection of oral cancer is highly recommended [45]. In a survey of dental students, recent graduates, and dental practitioners in the United Arab Emirates, it was found that participants did not exhibit a satisfactory diagnostic capability in recognizing mucosal changes consistent in oral cancer presentations [46]. Additionally, there is a need for increased and improved educational methods for undergraduate dental students to identify oral cancer and premalignant lesions [46].

Smoking cessation counselling from health care providers can assist smokers in quitting [47, 48]. In a previous study on dentist views on smoking cessation counseling, it was found that 50% of dentists believe that they have a role in smoking cessation, but lack of training and time were reported barriers [49]. Dentists who implement smoking cessation programs in their offices can achieve cessation rates up to 10–15% each year amongst patients who use smokeless tobacco or smoke [48]. When considering alcohol, dentists noted lack of knowledge and lack of referral resources as barriers to addressing alcohol cessation amongst their patients [50]. In this study, almost half of dentists were interested in receiving greater training on alcohol and smoking cessation programs and over 75% had an interest in attending a continuing education course on oral cancer detection and counseling. Given that dentists recognize the importance of risk factor counseling, it may be an important topic to be addressed in future continuing education courses [50]. Interestingly, there were variations with regards to remuneration for risk factor counselling; over half of dentists were unsure if they could be compensated for counselling, whereas 20% were billing for it. Standardization of billing practices to encourage dentists to perform oral cancer screening is going to be particularly important as there is renewed interest in providing government-funded dental care to those most in need.

In Ontario, over a quarter of patients reported not visiting a dentist in the last year [51]. Socioeconomic status, self-reported oral health, and general health behaviors are known to be associated with dental care use and overall oral health [51–54]. Indigenous status, low educational attainment, smoking status, low household income, and poor oral health were found to be associated with decreased likelihood of visiting the dentist, and only visiting for emergency care [51]. These factors are also risk factors for oral cavity cancer; it is vital that those who are least likely to use dental care are also screened for oral cavity cancer when they are assessed. However, dental care in Canada is not publicly funded, serving as a barrier for patients who may be most at risk for oral cavity cancer. A new dental coverage plan in Canada is set to be in place by 2025 and will aid in providing dental care and exams for low-income families. Families earning below $90,000 CAD and lacking dental insurance are proposed to be eligible for coverage and those earning below $70,000 CAD would have some dental fees fully covered [55]. However, it is still unclear what will be covered and how this will be implemented. While it was found that compensation or insurance status did not influence dentists’ decision to perform oral examinations, such coverage can allow for more patients to be captured with oral cavity screening. With the expansion of publicly funded dental care in Canada, it is critical to consider the incorporation of oral cancer screening in dental practices for the public.

This study has several limitations. Firstly, given that it was a cross-sectional study, it is subject to recall bias. This could possibly lead to an under or over reporting of the variables in the survey. While the survey was developed using similar studies as a reference guide and tested amongst a convenient sample of dentists, the specific internal consistency of the survey was not measured. Nonetheless, during the trial period of the survey, we did assess face validity and ease of interpretation both of which were well received by our testing sample of dentists. Secondly, the experiences of dental professionals with academic affiliations were investigated. While this could limit the generalizability of the results to dental professionals working in community or rural settings, dentists employed within community settings (small and medium-sized cities) participated in the survey and 12% of participating dentists were employed in cities with populations below 99,000. While our data may only be generalizable to dentists in a large metropolitan city in Canada, it is unlikely that dental practices significantly differ in smaller communities and other provinces given similar training and funding. Nonetheless, the results must be interpreted with caution. Also, while several attempts were made to recruit participants (maximal allowable by research ethics approval), there was a relatively low response rate (14%), limiting the generalizability of our study. However, this response rate is in keeping with that of medical professionals. There is significant variation in response rates across different provider types, and amongst dentists and other healthcare professionals response rates have been steadily declining to as low as 2% [56–58]. Also, the results are reflecting of the current billing and healthcare coverage in Canada and may not be generalizable to different healthcare system models. Even with the limitations in generalizability, there is valuable data that can be used to develop future studies with mixed methods approaches. Thirdly, the practices of both general dentists and dental specialists were captured, and subspeciality of practice was not specified within the survey. Screening practices may defer between general dentists and specialists depending on conditions they most frequently encounter.

This study was able to identify barriers to oral cavity cancer screening among dental professionals and the results of this study can be utilized to inform future additional larger studies. Future studies should investigate whether dental professionals believe that cancer screening is within the realm of a dentist’s scope of practice and whether dentists seek out continuing education courses if they feel unqualified to detect oral cancer. Additionally, future studies should seek to identify means to improve cancer screening in dental offices and to identify modifiable factors that prevent such practices. Prior to proceeding to such a study, which would likely require a very large survey administrated through dental societies, a deeper dive is required into what the limitations are at the dentist’s office. The best way to capture this data would be through a qualitative study with semi-structured interviews which can then inform future survey designs. Future studies should ideally include both academic and community dental professionals, best captured through dental societies, although there may still be a slight bias towards academic dental professionals even in that setting. Also, future studies should assess both oral cavity cancer and oropharyngeal cancer, particularly given the rising incidence of oropharynx cancer and the role of the dentist and dental hygienist in assessing the oropharynx.

## Conclusion

Dental professionals in Ontario have a good knowledge base for risk factors of oral cavity cancer and are confident in their ability to screen for oral cavity cancer. Almost all dentists perform oral cancer screening on all patients, regardless of their insurance status. Lack of time was the greatest reported barrier for oral cavity screening and compensation did not influence dentists’ decision to perform screening. Very few dentists provided risk factor counselling and were aware of smoking and alcohol cessation resources for their patients. Future continuing education events should address these gaps in knowledge particularly as there is increased interest in providing publicly funded dental care to those most at need.

## Supplementary Information

**Additional file 1.**

## Data Availability

The datasets used and/or analysed during the current study available from the corresponding author on reasonable request.
