# Supplementary material for: Oral cancer screening knowledge and practices among dental professionals at the University of Toronto
Source: BMC Oral Health. 2023 May 31;23:343. doi: 10.1186/s12903-023-03062-3 (PMC10230684; doi:10.1186/s12903-023-03062-3)
Supplement: Supplementary file 1 — Additional file 1. [file 12903_2023_3062_MOESM1_ESM.docx]

**Demographics**

What is your occupation?

1. Dentist (including dental specialists)
2. Dental hygienist

How do you identify?

1. Female
2. Male
3. Other
4. Prefer not to say

How many years have you been in practice?

1. < 5 years
2. 6-10 years
3. 11-20 years
4. 20+ years

Where did you complete your first dental degree?

1. Canada
2. Other:

What is the setting of your practice?

1. Small population centers (1,000 - 29,999)
2. Medium population centers (30,000 - 99,999)
3. Large urban population centers (100,000+)

**Knowledge of risk factors**

What are the TWO most common site of oral cancer? Select two answers.

1. Labial mucosa
2. Ventral surface of oral tongue
3. Floor of mouth
4. Mandibular alveolus
5. Maxillary alveolus
6. Retromolar triangle
7. Hard palate

Which of the following are the three HIGHEST RISK factors associated with oral cancer? Select three answers.

1. Smoking
2. Chew/snuff/snus/dip/betel nut
3. Alcohol
4. Diet
5. Human papillomavirus vaccine
6. Age
7. Sun exposure
8. Prior history of cancer
9. Other:

What is the most common clinical presentation of early symptoms of oral cancer?

1. Swelling at the site
2. Pain
3. Non-healing ulcer
4. White lesion
5. Red lesion
6. Enlarged lymph nodes
7. Other:

**Current practices**

Who performs oral cancer screening in your clinic?

1. Dentist (including dental specialists)
2. Dental hygienists
3. Both dentists and dental hygienists

How often do you perform intra/extra oral exams to screen for oral cancer at INITIAL patient appointments?

1. With every patient
2. With 50% or more of patients
3. With less than 50% of patients
4. Very rarely
5. Never

How often do you perform intra/extra oral exams to screen for oral cancer at FOLLOW UP patient appointments?

1. With every patient
2. With 50% or more of patients
3. With less than 50% of patients
4. Very rarely
5. Never

How often do you perform intra/extra oral exams to screen for oral cancer in ASYMPTOMATIC patients?

1. With every patient
2. With 50% or more of patients
3. With less than 50% of patients
4. Very rarely
5. Never

How often do you discuss risk factors of oral cancer with your patients?

1. With every patient
2. With 50% or more of patients
3. With less than 50% of patients
4. Very rarely
5. Never

If you have a patient with a pre-malignant lesion, such as mild dysplasia, at what interval do you schedule follow-up appointments?

1. ≤ 6 weeks
2. 3 months
3. 4 months
4. 6 months
5. 12 months
6. N/A: I never screen for oral cavity cancer
7. Other:

What influences your decision to perform an oral cancer screening examination? Check all that apply.

1. Patient symptoms
2. Age
3. Medical history
4. Presence of risk factors
5. None of the above. I perform oral cancer screening examinations on all my patients.
6. Other:

Which risk factors prompt you to perform an examination? Select all that apply.

1. Smoking
2. Chew/snuff/snus/dip/betel nut
3. Alcohol
4. Diet
5. Human papillomavirus vaccine
6. Age
7. Sun exposure
8. Prior history of cancer
9. Other:
10. None of the above. I perform oral cancer screening examinations on all my patients.

How often do you use each of the following components of an exam to detect oral cancer (in every patient, in more than 50% of patients, in less than 50% of patients, very rarely)?

1. Imaging: X ray
2. History
3. Intra oral visual examination
4. Extraoral visual examination
5. Oropharynx visual examination
6. Neck palpation

*Extra oral examination*

When performing an extra oral examination, how often do you… (always, usually, sometimes, rarely, never)

1. Inspect the skin, eyes, lips
2. Palpate the lymph nodes of the head and neck region
3. Palpate the salivary glands
4. Palpate the TMJ upon opening and closing of the jaw?

*Intra oral examination*

When performing an intra oral examination, how often do you… (always, usually, sometimes, rarely, never)

1. Visually inspect the lips and vermillion border
2. Palpate the tongue
3. Visually inspect the oral cavity
4. Palpate the lips and cheeks
5. Palpate floor of mouth
6. Check salivary gland function
7. Palpate tonsils
8. Inspect hard and soft palate

How much time do you spend performing an intra/extra oral examination?

1. < 1 minute
2. 1-3 minutes
3. 3-5 minutes
4. >5 minutes

If you do not always perform comprehensive intra/extra oral examinations, what is the reason? Select all that apply.

1. Not trained enough
2. Not necessary for all patients
3. Time constraints
4. Not effective
5. Other

How do you manage patients with suspected oral cancer?

1. Imaging (X ray)
2. Photo documentation
3. Biopsy
4. Direct referral to specialist
5. Ask patients to seek specialist advice on their own
6. Follow up at later appointment
7. Ask patient to monitor the lesion
8. Other: please specify

How often do you counsel patients on risk factor management?

1. In every patient
2. In more than 50% of patients
3. In less than 50% of patients
4. Very rarely
5. Never

Are you familiar with resources for smoking cessation and alcohol abuse programs to provide to patients?

1. Yes
2. No

Do you have an interest in receiving greater training on tobacco and alcohol cessation programs for patients?

1. Yes
2. No

**Attitudes**

How confident are you that your current knowledge on oral cancer is up to date?

1. Very confident
2. Confident
3. Satisfactory
4. Not confident

How would you rate your knowledge on oral cancer detection and prevention?

1. Very good
2. Good
3. Satisfactory
4. Poor
5. Very poor

Do you believe you received sufficient training to detect lesions suspicious for oral cancer?

1. Yes
2. No

How confident are you in your ability to detect oral cancer?

1. Very confident
2. Confident
3. Satisfactory
4. Not confident

How confident are you in your ability to perform a comprehensive oral examination?

1. Very confident
2. Confident
3. Satisfactory
4. Not confident

When is the last time you attended a continuing education course which discussed screening or management of oral cancer?

1. < 12 months
2. 2-5 years
3. 5+ years
4. Never

Do you have interest in attending continuing education courses on the detection and counselling of oral cancer?

1. Yes
2. No

Do you believe that the screening for oral cancer through visual examination is an effective method for its early detection?

1. Yes
2. No

Which of the following, if any, are barriers to performing intra/extra oral exams routinely in your practice? Select all that apply

1. Someone else in the office performs the exam
2. Lack of knowledge and/or skills to perform the exam
3. Lack of time
4. Concerns about patient compliance
5. Concern about remuneration for services
6. Other

Of the barriers, what do feel is the biggest barrier to performing these exams? (Free text)

**Reimbursement**

Are you able to bill insurance companies or the patient for performing intra/extra oral examinations for oral cancer?

1. Yes
2. No

When billing for a routine examination at an initial appointment, does the fee include the oral cancer screening exam?

1. Yes
2. No
3. I am not sure

When billing for a routine examination at a recall appointment, does the fee include the oral cancer screening exam?

1. Yes
2. No
3. I am not sure

Do your screening practices differ depending on whether the patient has private health insurance or is insured through government services?

1. Yes
2. No

Would you perform oral examinations more often if you were paid?

1. Yes
2. No
3. Not applicable. I perform oral cancer screening on all my patients.

Can you bill for smoking/alcohol cessation?

1. Yes
2. No
3. I am not sure

Would you counsel patients on risk factor management more often if you were paid?

1. Yes
2. No
3. Not applicable. I counsel all my patients on risk factor management.
